# Supplementary material for: Screening of C. auris among Candida isolates from various tertiary care institutions in Lahore by VITEK 2 and real time PCR based molecular technique
Source: PLoS One. 2023 Oct 24;18(10):e0293390. doi: 10.1371/journal.pone.0293390 (PMC10597507; doi:10.1371/journal.pone.0293390)
Supplement: Attachment — Submitted filename: Comments on Manuscript Number PONE-D-23-13143.pdf [file pone.0293390.s002.pdf]

Reviewer Recommendation and Comments for Manuscript Number PONE-D-23-13143

**Article Title:**	Molecular Identification of *Candida auris* by VITEK 2 and ITS-1 and ITS-2 regions of rDNA to early detect and limit infection in hospital setting of Lahore, Pakistan
**Comments & Recommendations:**	The title of the article is not suitable and it should be revised because the author did not identify any single isolate as *Candida auris* by any technique. However, she tried to screen the C. auris in the samples. One of the proposed title in my opinion is, **"Screening of *C. auris* among *Candida* isolates from various tertiary care institutions in Lahore by VITEK 2 and real time PCR based molecular technique"**.
**Methodology:**	The author collected *Candida* isolates from different tertiary care institution of Lahore and screened them for the presence of C. auris by testing Fluconazole @ 25µg.
**Comments & Recommendations:**	It may be mentioned here that Minimum Inhibitory Concentration for *C. auris* is known to be ≥32µg, therefore if any one desired to screen on the basis of fluconazole resistance then the used concentration should be appropriate. Moreover, the best antifungal activity against C. auris is known by the use of Echinocandins (Please refer Sanyaolau *et al*., 2022; https://doi.org/10.3947/ic.2022.0008). Therefore, the author is advised to screen Echinocandins rather than Fluconazole. More references may be found if properly searched.
**Results:**	The author mentioned that she screened 636 *Candida* isolates and for fluconazole resistance and found resistance in 248 (38.9%) isolates, and identify 87 isolates as different *Candida* species on the basis of VITEK 2 ID Compact System and performed RT-PCR on selected isolates (n=100).
**Comments & Recommendations:**	The author did not mention the criteria for selecting 87 and 100 isolates rather than testing all 248 fluconaozole resistant *Candida* isolates. However, she is claiming that C. auris could not be identify from collected samples.<br>It is suggested that to author to:<br>• Screen isolates resistant isolates using echinocandins or any other or appropriate concentration of fluconazole antifungal.<br>• Test all resistant *Candida* isolates or all collected isolates using VITEK 2 ID Compact System and RT-PCR.<br>• The author is also advised to use any control sample (positive control) for comparing the amplification on RT-PCR.

**Statistical Analysis:**	The author did not perform any statistical analysis
**Comments & Recommendations:**	The author is advised to”<br>• Mention the name of institutes form where the samples were collected with exact numbers and gender.<br>• The data may be classified into various institutes/hospitals and gender (male & female).<br>• The data may analyze statistically in terms of means, standard error and standard deviation.
**Overall Comments & Recommendations:**	The manuscript may be sent to author for major revision. The manuscript may be accepted after incorporating all the comments.
