# Supplementary material for: Screening of C. auris among Candida isolates from various tertiary care institutions in Lahore by VITEK 2 and real time PCR based molecular technique
Source: PLoS One. 2023 Oct 24;18(10):e0293390. doi: 10.1371/journal.pone.0293390 (PMC10597507; doi:10.1371/journal.pone.0293390)
Supplement: Attachment — Submitted filename: Response to Reviewers.docx [file pone.0293390.s003.docx]

**RESPONSE TO REVIEWERS**

Dear Editor and Reviewers,

Thank you, for your detailed and valuable Response to our manuscript. We believe that the points you have raised will improve the quality of our article. Please find the response to each comment below in this document.

**Comments from the Editor:**

1- Please ensure that your manuscript meets PLOS ONE's style requirements, including those for file naming. The PLOS ONE style templates can be found at

<https://journals.plos.org/plosone/s/file?id=wjVg/PLOSOne_formatting_sample_main_body.pdf> and 
<https://journals.plos.org/plosone/s/file?id=ba62/PLOSOne_formatting_sample_title_authors_affiliations.pdf>

***Response:*** *The title page and the rest of the manuscript have been formatted according to the requirement and the style provided.*

**2.** We suggest you thoroughly copyedit your manuscript for language usage, spelling, and grammar. If you do not know anyone who can help you do this, you may wish to consider employing a professional scientific editing service.

Whilst you may use any professional scientific editing service of your choice, PLOS has partnered with both American Journal Experts (AJE) and Editage to provide discounted services to PLOS authors. Both organizations have experience helping authors meet PLOS guidelines and can provide language editing, translation, manuscript formatting, and figure formatting to ensure your manuscript meets our submission guidelines. To take advantage of our partnership with AJE, visit the AJE website (<http://learn.aje.com/plos/>) for a 15% discount off AJE services. To take advantage of our partnership with Editage, visit the Editage website ([www.editage.com](http://www.editage.com/)) and enter referral code PLOSEDIT for a 15% discount off Editage services.  If the PLOS editorial team finds any language issues in text that either AJE or Editage has edited, the service provider will re-edit the text for free.

Upon resubmission, please provide the following:

a) The name of the colleague or the details of the professional service that edited your manuscript.

b) A copy of your manuscript showing your changes by either highlighting them or using track changes (uploaded as a *supporting information* file).

c) A clean copy of the edited manuscript (uploaded as the new *manuscript* file).

***Response:*** *a-“Grammerly” was used to check the grammer, spelling, lagguage usage, wordiness, style, and punctuation.*

*b-“Quillbot” was used for paraphrasing and to enhance the writing.*

*c-Moreover, the language editing, spelling, and grammer check were additionally carried out by all the authors themselves.*

3-Thank you for stating the following financial disclosure: "no"

At this time, please address the following queries:

a) Please clarify the sources of funding (financial or material support) for your study. List the grants or organizations that supported your study, including funding received from your institution.
b) State what role the funders took in the study. If the funders had no role in your study, please state: “The funders had no role in study design, data collection and analysis, decision to publish, or preparation of the manuscript.”
c) If any authors received a salary from any of your funders, please state which authors and which funders.
d) If you did not receive any funding for this study, please state: “The authors received no specific funding for this work.”

Please include your amended statements within your cover letter; we will change the online submission form on your behalf.

***Response:***

*a) The present study has not received any grant or any organizational funding. However, the present study has support from the institution, University of Health Sciences, Lahore, Pakistan.*

*b) The institution provided supported the study, by providing the consumables for the present study.*

*“The funders had no role in study design, data collection and analysis, decision to publish, or preparation of the manuscript.”*

*c) No, the author has not received any funding from the institution or funder.*

*However, Dr. Sidrah Saleem and Dr. Muhammad Imran are working as professors and Associate professors respectively in the Department of Microbiology, University of Health Sciences, Lahore, Pakistan. Dr Ali Amar is working as a lecturer in the Department of Human Genetics and Molecular Biology, University of Health Sciences, Lahore, Pakistan.*

*d) Other than consumables “The authors received no specific funding for this work.”*

*The statement has been included in the cover letter.*

4. We note that you have stated that you will provide repository information for your data at acceptance. Should your manuscript be accepted for publication, we will hold it until you provide the relevant accession numbers or DOIs necessary to access your data. If you wish to make changes to your Data Availability statement, please describe these changes in your cover letter and we will update your Data Availability statement to reflect the information you provide.

***Response:*** *The data is provided in the form of supporting information (separate file).*

5. Please amend your authorship list in your manuscript file to include author "Ali Amar".

***Response:***

*The name of the author has been added.*

6. Please amend either the abstract on the online submission form (via Edit Submission) or the abstract in the manuscript so that they are identical.

***Response:*** *done*

7. We note you have included a table to which you do not refer in the text of your manuscript. Please ensure that you refer to Table 2 in your text; if accepted, production will need this reference to link the reader to the Table.

***Response:*** *Table no 2 has been added in the text.*

**Reviewers' comments:**

Reviewer's Responses to Questions

**Comments to the Author**

1. Is the manuscript technically sound, and do the data support the conclusions?

The manuscript must describe a technically sound piece of scientific research with data that supports the conclusions. Experiments must have been conducted rigorously, with appropriate controls, replication, and sample sizes. The conclusions must be drawn appropriately based on the data presented.

Reviewer #1: No

Reviewer #2: Partly

***Response:*** *conclusion added*

2. Has the statistical analysis been performed appropriately and rigorously?

Reviewer #1: I Don't Know

Reviewer #2: No

***Response:*** *Added*

3. Have the authors made all data underlying the findings in their manuscript fully available?

The [PLOS Data policy](http://www.plosone.org/static/policies.action#sharing) requires authors to make all data underlying the findings described in their manuscript fully available without restriction, with rare exception (please refer to the Data Availability Statement in the manuscript PDF file). The data should be provided as part of the manuscript or its supporting information, or deposited to a public repository. For example, in addition to summary statistics, the data points behind means, medians and variance measures should be available. If there are restrictions on publicly sharing data—e.g. participant privacy or use of data from a third party—those must be specified.

Reviewer #1: Yes

Reviewer #2: No

***Response:*** *Provided as separate “supporting information”.*

4. Is the manuscript presented in an intelligible fashion and written in standard English?

PLOS ONE does not copyedit accepted manuscripts, so the language in submitted articles must be clear, correct, and unambiguous. Any typographical or grammatical errors should be corrected at revision, so please note any specific errors here.

Reviewer #1: Yes

Reviewer #2: Yes

***Response:*** *The reviewer’s response is positive.*

5. Review Comments to the Author

Please use the space provided to explain your answers to the questions above. You may also include additional comments for the author, including concerns about dual publication, research ethics, or publication ethics. (Please upload your review as an attachment if it exceeds 20,000 characters)

**Reviewer #1:**

The draft "Molecular Identification of Candida auris by VITEK 2 and ITS-1 and ITS-2 regions of rDNA to early detect and limit infection in hospital setting of Lahore, Pakistan" can be important if author do major revision and make necessary additions to draft. My specific comments are

1) Draft need minor writing check

***Response****: Done*

2) In support of table 2, author have not shown any data like drug sensitivity or tolerance by spotting isolates on drug plate.

***Response****: An antifungal drug i.e. Fluconazole was used to screen for the resistant isolates for further testing for Candida auris. Candida auris is known to be resistant to the Azole class of antifungal drugs.*

3) Dont know what author want to convey from RT-PCR data shown in figure, there should be a positive control in RT-PCR to show that PCR works

**Response**: *None of the Candida auris were screened out, that's why the figure is removed as it does not add useful information.*

4) Experiments were done with poor logic and proper controls are missing.

***Response****: Candida albicans (ATCC 10231) was used.*

**Reviewer #2: Reviewer Recommendation and Comments for Manuscript Number PONE-D-23-13143**

**Article Title:**

Molecular Identification of Candida auris by VITEK 2 and ITS-1 and ITS-2 regions of rDNA to early detect and limit infection in hospital setting of Lahore, Pakistan

Comments & Recommendations:

The title of the article is not suitable and it should be revised because the author did not identify any single isolate as Candida auris by any technique. However, she tried to screen the C. auris in the samples. One of the proposed title in my opinion is, “Screening of C. auris among Candida isolates from various tertiary care institutions in Lahore by VITEK 2 and real time PCR based molecular technique”.

***Response:*** *The title is changed according to the recommendation.*

**Methodology:**
The author collected Candida isolates from different tertiary care institution of Lahore and screened them for the presence of C. auris by testing Fluconazole @ 25μg.

Comments & Recommendations:

It may be mentioned here that Minimum Inhibitory Concentration for C. auris is known to be 32μg, therefore if any one desired to screen on the basis of fluconazole resistance, then the used concentration should be appropriate. Moreover, the best antifungal activity against C. auris is known by the use of Echinocandins (Please refer Sanyaolau et al., 2022; <https://doi.org/10.3947/ic.2022.0008>). Therefore, the author is advised to screen Echinocandins rather than Fluconazole. More references may be found if properly searched.

***Response:*** *The Fluconazole disc @ 25μg was used to screen for the Azole-resistant isolates of the Candida species (majority of the Candida species are sensitive to Fluconazole and Candida auris is resistant) that were at the second step, ruled out for the identification of the Candida auris by the PCR method.*

**Results:**
The author mentioned that she screened 636 Candida isolates and for fluconazole resistance and found resistance in 248 (38.9%) isolates and identify 87 isolates as different Candida species on the basis of VITEK 2 ID Compact System and performed RT-PCR on selected isolates (n=100).

Comments & Recommendations:

The author did not mention the criteria for selecting 87 and 100 isolates rather than testing all 248 fluconaozole resistant Candida isolates. However, she is claiming that C. auris could not be identify from collected samples.

It is suggested that to author to:

• Screen isolates resistant isolates using echinocandins or any other or appropriate concentration of fluconazole antifungal.

***Response:*** *As for the majority of the Candida species (Candida albicans, C. tropicalis, etc.), the drug of choice is Azoles. Candida auris is resistant to the Azole class of antifungal drugs, A Fluconazole disk of 25μg was used to screen the Azole-resistant isolates (that include Candida auris as well). Out of 248 fluconazole-resistant Candida isolates, 100 isolates were randomly selected for further screening for the Candida auris species.*

*Yes, Echinocandins are the first-line drug for the treatment of Candida auris. They are used for the treatment of Candia auris in the first place. In case the Echinocandin is used to screen the resistant Candida species, then the majority of the Candida species (Candida albicans, C. krusei, C. glabrata, C. auris) turned out sensitive and it becomes difficult to identify the resistant strains.*

*Moreover, as the sample size calculated for the present study is 85. So the university has facilitated the consumables to this sample size only.*

*Reference: regarding fluconazole resistance in Candida auris*

[*https://link.springer.com/article/10.1007/s00203-022-03159-3*](https://link.springer.com/article/10.1007/s00203-022-03159-3)

• Test all resistant Candida isolates or all collected isolates using VITEK 2 ID Compact System and RT-PCR.

***Response:*** *Yes, it is the most appropriate approach to screen Candida auris among all the Fluconazole-resistant Candida, but due to budget constraints and limited resources, VITEK 2 and RT-PCR was not performed on all of the resistant isolates. As mentioned earlier, no grant or funding is available for this study and only little possible support was provided in the form of consumables by the institution.*

*The present study utilizes VITEK 2 version 8.01, which is capable of correctly identifying the Candida auris. According to the statement given by the Company (they added Candida auris to their database in that version) and later on supported by many researchers, the updated version can correctly identify the Candida auris species. The problem of misidentification was with the previous software, in which if Candida haemolonii / Candida duobushaemulonii were identified then the identification had to be confirmed by the molecular methods. We have performed our study on the most recent and updated version of VITEK 2 and none of our isolates identified as Candida haemolonii / Candida duobushaemulonii. However, we have also performed realtime PCR molecular methods to double-check our results.*

*References: 1-* [*https://www.cdc.gov/fungal/candida-auris/identification.html*](https://www.cdc.gov/fungal/candida-auris/identification.html)

2- [*https://www.chicagohan.org/documents/14171/427345/Algorithm+for+C+auris+identification.pdf/3ff08393-4361-9b42-6ac4-642f8c316ffe?t=1611162101069*](https://www.chicagohan.org/documents/14171/427345/Algorithm+for+C+auris+identification.pdf/3ff08393-4361-9b42-6ac4-642f8c316ffe?t=1611162101069)
3- [*https://pubmed.ncbi.nlm.nih.gov/31413079/*](https://pubmed.ncbi.nlm.nih.gov/31413079/)
4- [*https://link.springer.com/article/10.1007/s00203-022-03159-3*](https://link.springer.com/article/10.1007/s00203-022-03159-3)

• The author is also advised to use any control sample (positive control) for comparing the amplification on RT-PCR.

**Response:** *Candida albicans (ATCC 10231) was used as a control in the study.*

*The species specificity of the primers CAURF and CAURR for C. auris used in the present study were indicated by BLAST searches as they showed complete sequence identity only with C. auris strains. The same sequence was used in the previous study by Khan et al.,2018*

*(*[*https://journals.plos.org/plosone/article/file?id=10.1371/journal.pone.0195743&type=printable*](https://journals.plos.org/plosone/article/file?id=10.1371/journal.pone.0195743&type=printable) *). This reference study has also not used the ATCC control of Candida auris because it is not readily available, high priced, and because of biosafety and biosecurity concerns.*

**Statistical Analysis:** The author did not perform any statistical analysis.

Comments & Recommendations:

The author is advised to:

• Mention the name of institutes form where the samples were collected with exact numbers and gender.

***Response****: The required added as Table no 02.*

• The data may be classified into various institutes/hospitals and gender (male & female).

**Response:** *The required data is added.*

• The data may analyze statistically in terms of means, standard error and standard deviation.

**Response:** *As most of the data is categorical, determination of mean, standard error, or standard deviation is not possible. However, regarding all the numerical data, the required statistical analysis is added.*

Overall Comments & Recommendations:

The manuscript may be sent to author for major revision. The manuscript may be accepted after incorporating all the comments.

6. PLOS authors have the option to publish the peer review history of their article ([what does this mean?](https://journals.plos.org/plosone/s/editorial-and-peer-review-process#loc-peer-review-history)). If published, this will include your full peer review and any attached files.

If you choose “no”, your identity will remain anonymous but your review may still be made public.

**Do you want your identity to be public for this peer review?**

For information about this choice, including consent withdrawal, please see our [Privacy Policy](https://www.plos.org/privacy-policy).

Reviewer #1: No

Reviewer #2: No
